# Genome-wide association study for backfat thickness in Canchim beef cattle using Random Forest approach

**DOI:** 10.1186/1471-2156-14-47

**Published:** 2013-06-05

**Authors:** Fabiana Barichello Mokry, Roberto Hiroshi Higa, Maurício de Alvarenga Mudadu, Andressa Oliveira de Lima, Sarah Laguna Conceição Meirelles, Marcos Vinicius Gualberto Barbosa da Silva, Fernando Flores Cardoso, Maurício Morgado de Oliveira, Ismael Urbinati, Simone Cristina Méo Niciura, Rymer Ramiz Tullio, Maurício Mello de Alencar, Luciana Correia de Almeida Regitano

**Affiliations:** 1 Department of Genetics and Evolution, Federal University of São Carlos, Rodovia Washington Luiz, km 235, PO BOX 676, 13565-905, São Carlos, Brazil; 2 Embrapa Agricultural Informatics, Avenida André Tosello, 209, PO BOX 6041, 13083-886, Campinas, Brazil; 3 Embrapa Southeast Livestock, Rodovia Washington Luiz, km 234, PO BOX 339, 13560-970, São Carlos, Brazil; 4 Department of Animal Science, Federal University of Lavras, PO BOX 3037, 37200-00, Lavras, Brazil; 5 Embrapa Dairy Cattle, Rua Eugênio do Nascimento, 610, 36038-330, Juiz de Fora, Brazil; 6 Embrapa Southern Region Animal Husbandry, BR 153, km 603, PO BOX 242, 96401-970, Bagé, Brazil; 7 Department of Exact Science, São Paulo State University, PO BOX 53453, 14884-900, Jaboticabal, Brazil

**Keywords:** Bovine, Lipid metabolism, Machine learning, Single nucleotide polymorphism (SNP), Subcutaneous fat, Tropical composite cattle

## Abstract

**Background:**

Meat quality involves many traits, such as marbling, tenderness, juiciness, and backfat thickness, all of which require attention from livestock producers. Backfat thickness improvement by means of traditional selection techniques in Canchim beef cattle has been challenging due to its low heritability, and it is measured late in an animal’s life. Therefore, the implementation of new methodologies for identification of single nucleotide polymorphisms (SNPs) linked to backfat thickness are an important strategy for genetic improvement of carcass and meat quality.

**Results:**

The set of SNPs identified by the random forest approach explained as much as 50% of the deregressed estimated breeding value (dEBV) variance associated with backfat thickness, and a small set of 5 SNPs were able to explain 34% of the dEBV for backfat thickness. Several quantitative trait loci (QTL) for fat-related traits were found in the surrounding areas of the SNPs, as well as many genes with roles in lipid metabolism.

**Conclusions:**

These results provided a better understanding of the backfat deposition and regulation pathways, and can be considered a starting point for future implementation of a genomic selection program for backfat thickness in Canchim beef cattle.

## Background

Beef cattle production in Brazil is based on several breeds, depending on the geography and climate of a given area. Breeds based on *Bos taurus* are commonly raised as livestock for beef in the South of Brazil, but in most parts of the country, beef cattle production is based on *Bos indicus* (zebu) breeds raised on natural pastures. A good description of Brazilian beef cattle production was recently published [1]. Zebu breeds are considered highly adapted to the tropical environment in Brazil [2-5], but they are known for their lower meat quality in certain aspects, such as tenderness, palatability, and marbling [6-10], and for their lower reproduction efficiency [11,12] when compared to *Bos taurus*. The Canchim (3/8 zebu + 5/8 Charolais) breed was developed in the early 1960’s in Brazil [13] with the intention of combining fitness traits from zebu to the higher reproduction efficiency and meat quality from the Charolais breed.

Although the Canchim breed has fared well when raised on natural pastures in Brazil, some carcass traits have still remained inferior when compared to *Bos taurus*. One such trait is backfat thickness, which has been a concern for Canchim producers, and for the beef cattle industry in general, due to its low fat deposition in animals raised on pasture (1.90mm ± 0.77) [14]. Improvement of this trait in Canchim beef cattle using traditional selection techniques has had limited success because of its relatively low heritability (0.23) [14], and because it is measured late in an animal’s life. Most studies available in the literature regarding backfat thickness have been conducted on animals raised in feedlot systems, which permits earlier ultrasound measurements, and has also shown moderate to high heritabilities [15-19], thereby allowing traditional selection techniques under these conditions to be more successful than compared to the Canchim breed.

In attempts to improve meat quality, previous studies have focused on the identification of candidate markers associated with meat quality traits, as well as backfat thickness, in Canchim and other *Bos indicus* × *Bos taurus* crosses in Brazil [20-24]. However, these have had limited success, particularly in response to markers on the *DDEF1* and *LEP* genes [20,23]. Therefore, the identification of genetic markers linked to backfat thickness by novel methodologies is an important strategy for genetic improvement of carcass and meat qualities. One recently developed approach relies on examining how SNPs (single nucleotide polymorphisms) are associated with these qualitative traits [25]. More specifically, this method has been used successfully in studies that examined fat-related traits, such as intramuscular fat percentage, marbling, rib fat, backfat thickness and rump fat depth [26-35]. By the use of high-density SNP panel assays for different breeds and crosses, these studies have collectively found such traits associated with regions on nine bovine chromosomes (6, 15, 17, 20, 21, 24, 25, 26, and 28) [27,28,32,35]. However, another study suggested that some of the effects attributed to each SNP can show variation based on the breed’s origin, resulting from variation in indicine and taurine-indicine composite cattle [35], thereby justifying the investigation of SNPs based on the breed of interest.

A previous study using high-density SNP panel has associated 100 SNPs to backfat thickness in a Canchim population using an approach that selected animals with extreme phenotypes for genotyping [33]. Those SNPs were located on several bovine autosomes, and from them, the authors further investigated and validated two regions on chromosome (chr) 14 associated with backfat thickness, where the haplotypes were responsible for 0.24% to 1.1% of the phenotypic variance for this trait.

Although these results are useful, it is well known that quantitative traits are polygenic as each SNP may account for only a small part of the phenotypic variance, therefore joint analysis of many SNPs has become a more interesting strategy [36,37]. This, however, exacerbates the ‘large p, small n’ problem faced by genome-wide studies, which means that there is a small number of phenotypes (n) to predict a large number of SNP (p) effects [38].

One solution to this problem is through the use of Random Forest, a machine learning algorithm capable of dealing with certain datasets for building model independent classification and/or regression problem predictors [39]. Specifically, it embeds a procedure of accounting for predictor variable importance, which results in a score that can be used for prioritizing variables (SNPs), similar to p-values from statistical tests [40-42]. Because of these features, the variable importance of the random forest method has been recognized as an useful methodology for genome-wide association studies [43].

Considering all of the above, the objectives of this study were to identify SNPs associated with backfat thickness in Canchim beef cattle using the random forest approach for genome-wide association studies, to shed insight on potential genes associated with this trait, and to discover potential SNPs for future implementation of genomic selection (GS). The set of SNPs identified by this methodology explains as much as 50% of the deregressed estimated breeding value variance associated with the observed phenotype. These results intend to provide a better understanding of the backfat deposition and regulatory pathways, and to enable the use of the identified SNPs in validation studies for genomic selection.

## Methods

### *Animals and phenotypes*

Animals used in this study were part of the Canchim Breeding Association from seven herds located in two Brazilian states (São Paulo and Goiás). This research is in agreement with the ethical principles of animal experimentation of Embrapa Southeast Livestock Ethical Committee of Animal Use (CEUA-CPPSE), and has been performed with the approval of CEUA-CPPSE under protocol number 02/2009. An initial sample of 987 animals (males and females) was evaluated for backfat thickness by ultrasound *in vivo* over the 12th rib around the age of 18 months. All animals evaluated were born between 2003 and 2005 and raised on natural pastures.

These 987 animals had the estimated breeding value (EBV) predicted by restricted maximum likelihood using the MTDFREML software [44]. The animal model included fixed effects of contemporary group (sex, year, herd, and genetic group) and age at measurement as a linear covariate, the additive genetic effect and error were included as random effects. From these animals, a sample of 400 was selected considering: EBV, accuracy, family size, and proportion between males (196) and females (204). These 400 animals were offspring of 50 different sires (with 1 to 30 offspring per sire).

### Genotyping and SNPs quality control

The selected 400 animals were genotyped using the BovineHD BeadChip (Illumina Inc., San Diego, CA). The quality control filters included call rate (< 0.90) for samples and SNPs, minor allele frequency (MAF < 0.01), and heterozygosity (< 3 standard deviations). After quality control processing, 396 animals and 708,641 SNPs with an average call rate higher than 0.99 remained in the study.

### Genome-wide association analysis

Genome-wide association (GWA) analysis was performed on deregressed EBVs (dEBV) [45], which takes into account the pedigree matrix, estimated heritability (0.16, data not shown), EBVs, and EBV's accuracies obtained by the same animal model described above. For the estimation of dEBVs the data set was enhanced with data collected from animals born between 2005 and 2008 totaling 1,648 individuals with phenotypes for backfat thickness, with 6,801 animals in the pedigree matrix.

Association of SNPs to dEBVs was undertaken by a random forest package [46] available in the R-project software [47]. The association analysis was composed of a two-step procedure. In the first step, the SNPs with the highest 1% importance score by chromosome were selected, and in the second step, the outcome set of SNPs from the first step was re-analyzed disregarding the chromosome classification, and the SNPs with the highest 1% importance score were selected. For the association analysis, the missing genotypes were imputed by the näive method provided in the random forest package (which imputes column median values for missing genotypes), the number of trees to grow and the number of randomly selected candidate SNPs at each split were set to 5,000 and 10% from the SNPs being evaluated, respectively. This procedure was done using the 396 samples available.

Taking into account the unbalanced offspring range among sires, 10 subsamples consisting of 198 animals each were also analyzed in the same two-step process as previously described. The 10 subsamples were selected as follows: i) The first animal was chosen at random from the 396 genotyped animals; ii) The next animal was selected based on the lowest relationship with the previous selected animal, but most representative from the rest of the genotyped animals; and iii) Step ii was repeated until 198 animals were selected.

Two approaches were considered for further SNP investigation among the results obtained by the random forest analysis. One approach selected the SNPs in common among the analysis with the 396 animals and the 10 subsamples, called the Common SNPs strategy. Another approach selected only the top 1% (importance score) from the analysis with 396 animals, called the Highest 1% SNPs.

Finally, after both sets of SNPs (Common SNPs and Highest 1% SNPs) had been selected, each set of selected SNPs were fitted into a final stepwise regression model using SAS/STAT software [48] to estimate the amount of variance explained by the selected SNPs in the data set (final model R^2^ values correspond to the dEBV variance explained by the model, which are reported in Table 1). For doing so, the SNPs were coded as 0, 1, and 2 for the AA, AB, and BB genotypes, respectively. In order to evaluate the significance of the results, a permutation test was conducted to estimate the bias associated with the R^2^ obtained from the stepwise regression analysis. In the permutation test, the dEBV values were shuffled and then regressed to the same SNPs previously selected. The permutation test was repeated 1,000 times.

**Table 1:** Number of candidate and final SNPs selected through the Common SNP and Highest 1% SNP strategies

	**Candidate SNPs**	**Final Model SNPs**	**% dEBV variance**^**(1)**^	**Permutation R**^**2(2)**^
Common SNP	162	19	50.59%	0.00 ± 0.02
Highest 1% SNP	70	21	53.27%	0.00 ± 0.02

^1^*dEBV* deregressed estimated breeding value variance explained by the final fitting of SNPs. The % dEBV variance is the model R^2^ from the final analysis which fits all SNPs as fixed effects into a regression analysis.

^2^ Permutation R^2^: average values and standard deviations for R^2^ from 1,000 permutation tests.

### Candidate genes and pathways

A pathway analysis was conducted to characterize the genomic regions identified by the set of SNPs previously selected and to identify candidate genes influencing biological functions and pathways related to backfat thickness and fat-related traits.

The software fastPHASE version 1.4.0 [49] was used for reconstructing the haplotypes for each chromosome. Afterwards, the reconstructed haplotypes were analyzed by the software Haploview [50] (using default parameters) for estimating haplotype blocks and linkage disequilibrium (LD), which was calculated based on the squared correlation coefficient between SNP pairs (r^2^). Considering the extent of LD based on the overall average r^2^ (average r^2^ = 0.12 at a distance of 250Kb, data not shown), a window of 500Kb (SNP position ± 250Kb) surrounding each SNP previously selected by the stepwise regression was considered to define the region used for candidate gene discovery and pathway annotation.

The Cattle Genome Browser through the UMD 3.1 Cattle genome assembly [51], was used for visualization of the selected SNPs and surrounding areas for localization and identification of QTLs, genes, and other interesting genomic landmarks. Other databases, such as the NCBI BioSystems database [52], and Kyoto Encyclopedia of Genes and Genomes (KEGG) [53,54] were also used for pathway annotation to gain insight into the biological processes involved in backfat thickness deposition.

## Results

We performed regression analysis for both strategies (Common SNP and Highest 1% SNP), and the results were very similar in the final number of SNPs selected, and the percentages of dEBV variance explained by the final set of SNPs (Table 1) enabling the discussion to be focused on the set of 21 SNPs selected from the Highest 1% SNP strategy due to its higher % of dEBV variance explained. Also, the first five SNPs (rs133046994, rs137294146, rs109349988, rs136717249, rs134790147) in the regression model were the same and in the same order for both strategies. These first five SNPs were responsible for 34.13% of dEBV variance for backfat thickness.

As a precaution against spurious artifacts that can result from splitting small samples into training and validation datasets, this was not performed here. An alternative option is to use a permutation test, which calculates the probability of obtaining a value more extreme than or equal to the observed value of a test statistic by shuffling the data and recalculating the test statistic. The proper test statistic for multiple regression is the coefficient of multiple determination, R^2^[55].

A permutation test was carried out to evaluate the probability of bias associated with the R^2^ from the stepwise regression analysis (Table 1). The average R^2^ from 1,000 permutation tests was 0.00 ± 0.02 for the Highest 1% SNP strategy, showing that there is a small bias associated with the R^2^ from the stepwise regression analysis. However, this is very small when compared to the 53.27% obtained from the Highest 1% SNP strategy, and therefore reinforces the significance of the results presented in Table 1.

Table 2 shows the 21 SNPs selected by the stepwise regression, their chromosome, position, % of dEBV variation explained by the SNP, genes annotated within ± 250Kb, fat-related QTLs described in the current literature, and references. Table 3 shows a summary of pathway annotation using the genes within ± 250Kb from the 21 selected SNPs using the KEGG [53,54] pathway database.

**Table 2:** Summary of information available for the Highest 1% SNPs selected by the stepwise regression

**dbSNP**^**1**^	**Chr**^**2**^	**Position**	**% dEBV**^**3**^	**Genes**^**4**^**in ± 250Kb**	**Fat-related QTL**^**5**^	**QTL reference**
rs133046994	10	18129602	11.12	*THSD4, LRRC49*	SF, MS	[65,66]
rs137294146	1	132385787	9.41	*SOX14, CLDN18, DZP1L*	FT12R, IF	[29,71]
rs109349988	3	15814096	5.21	*KCNN3, EFNA3, EFNA4, DCST2, LOC100294774, PMVK, ADAR, CHRNB2, ADAM15, ZBTB7B, LOC100294857, DCST1, FLAD1, PYGO2, CKS1B, PBXIP1, SHC1, LOC100294894*	FT12R, MS	[66]
rs136717249	19	37969870	4.88	*B4GALNT2, GNGT2, ABI3, PHOSPHO1, ZNF652, NGFR, PHB, IGF2BP1, GIP*	OAC, PAC	[84,85]
rs134790147	13	20780821	3.51	*CCDC7, ARL5B, MGC152301, LOC100848675, LOC100847992*	FT12R	[29]
rs136287610	25	42678992	2.89	*FAM20C, LOC783396, LOC100300875, LOC100337322, PRKAR1B, PDGFA, PTCHD3, LOC783852, LOC783961*	FT12R	[29]
rs136393667	11	65619399	2.51	*LOC786621, LOC100139826, ETAA1*	FT12R, MS	[29]
rs41790889	16	990255	2.07	*OPTC, PRELP, FMOD, BTG2, LOC789413, LOC789394, CHI3L1, MYBPH, ADORA1, LOC100847554*	FT12R, MS	[29]
rs42126516	4	52535108	1.99	*TFEC, LOC100296613, TES*	MS	[29,97]
rs42021729	3	64737352	1.46		MS	[98]
rs137001098	8	95507919	1.46	*SMC2, LOC100337180*	MS	[29]
rs43341824	1	50110036	1.23	*LOC785980, ALCAM*	OAC, FT12R	[85,99]
rs41683753	13	33219105	1.04	*CACNB2, NSUN6, EPC1, LOC10084770*	PAC, MS	[29,85]
rs136348926	12	10043410	0.90	*LOC786945*		
rs109869647	3	13195543	0.72	*LOC100849046, LOC100848852, LOC784007, LOC783963*	MS	[66]
rs110833507	11	42856561	0.69	*LOC100296234, LOC100296682, BCL11A*		
rs42923911	2	12761205	0.57	*LOC787311, LOC100848878*		
rs135638125	10	18147174	0.55	*THSD4, LRRC49*	MS, SF	[65,66]
rs110607520	9	96622647	0.66	*SYTL3, TULP4, TMEM181, EZR, LOC781263, DYNLT1, RSPH3, LOC782714, TAGAP, LOC782637*	MS	[29]
rs110025080	9	11710300	0.52	*RIMS1*		
rs109697559	2	61906393	0.58	*LOC100847709, LOC100297008, LCT, UBXN4, MCM6, DARS, R3HDM1, MIR128-1*	MS	[29]

^1^ Reference SNP cluster report.

^2^ Chromosome in *B. taurus*.

^3^ % dEBV variance is the model R^2^ for each of the SNPs in the final analysis which fits all SNPs as fixed effects into a regression analysis.

^4^ Gene symbol.

^5^*SF* subcutaneous fat, *FT12R* fat thickness at the 12th rib, *IF* intramuscular fat, *FT* fat thickness, *MS* marbling score, *OAC* oleic acid content, *PAC* palmitoleic acid content.

**Table 3:** Summary of pathway description from the KEGG Pathway Database

**Global Pathway**	**Subpathway**	**Pathway**	**Gene**	**SNP**
Metabolism	Carbohydrate Metabolism	Galactose metabolism	*LCT*	rs109697559
		Amino sugar and nucleotide sugar metabolism	*CHI3L1*	rs41790889
	Lipid Metabolism	Glycerophospholipid metabolism	*PHOSPHO1*	rs136717249
	Metabolism of Terpenoids and Polyketides	Terpenoid backbone biosynthesis	*PMVK*	rs109349988
	Metabolism of Cofactors and Vitamins	Riboflavin metabolism	*FLAD1*	rs109349988
Genetic Information Processing	Replication and Repair	DNA replication	*MCM6*	rs109697559
	Folding, Sorting and Degradation	RNA degradation	*BTG2*	rs41790889
	Translation	Aminoacyl-tRNA biosynthesis	*DARS*	rs109697559
Environmental Information Processing	Signal Transduction	MAPK signaling pathway	*CACNB2, PDGFA*	rs41683753, rs136287610
		ErbB signaling pathway	*SHC1*	rs109349988
	Signaling Molecules and Interaction	Cell adhesion molecules (CAMs)	*CLDN18, ALCAM*	rs137294146, rs43341824
		Neuroactive ligand-receptor interaction	*CHRNB2, GIP, ADORA1*	rs109349988, rs136717249, rs41790889
		Cytokine-cytokine receptor interaction	*NGFR, PDGFA*	rs136717249, rs136287610
Cellular Processes	Cell Motility	Regulation of actin cytoskeleton	*EZR, PDGFA*	rs110607520, rs136287610
	Cell Growth and Death	Cell cycle	*MCM6*	rs109697559
		Apoptosis	*PRKAR1B*	rs136287610
	Cell Communication	Tight junction	*CLDN18*	rs137294146
		Focal adhesion	*SHC1, PDGFA*	rs109349988, rs136287610
		Gap junction	*PDGFA*	rs136287610
	Transport and Catabolism	Peroxisome	*PMVK*	rs109349988
Organismal Systems	Circulatory System	Cardiac muscle contraction	*CACNB2*	rs41683753
	Immune System	Leukocyte transendothelial migration	*EZR, CLDN18*	rs110607520, rs137294146
		Chemokine signaling pathway	*GNGT2, SHC1*	rs136717249, rs109349988
		Cytosolic DNA-sensing pathway	*ADAR*	rs109349988
		Natural killer cell mediated cytotoxicity	*SHC1*	
	Digestive System	Gastric acid secretion	*EZR*	rs110607520
		Carbohydrate digestion and absorption	*LCT*	rs109697559
	Nervous System	Glutamatergic synapse	*GNGT2*	rs136717249
		GABAergic synapse	*GNGT2*	rs136717249
		Cholinergic synapse	*GNGT2, CHRNB2*	rs136717249, rs109349988
		Dopaminergic synapse	*GNGT2*	rs136717249
		Serotonergic synapse	*GNGT2*	rs136717249
		Retrograde endocannabinoid signaling	*GNGT2, RIMS1*	rs136717249, rs110025080
		Synaptic vesicle cycle	*RIMS1*	rs110025080
		Neurotrophin signaling pathway	*SHC1, NGFR*	rs109349988, rs136717249
	Development	Axon guidance	*EFNA3, EFNA4*	rs109349988
	Endocrine System	Insulin signaling pathway	*SHC1, PRKAR1B*	rs109349988, rs136287610
Human Diseases	Cardiovascular Diseases	Hypertrophic cardiomyopathy (HCM)	*CACNB2*	rs41683753
		Arrhythmogenic right ventricular cardiomyopathy (ARVC)	*CACNB2*	rs41683753
		Dilated cardiomyopathy (DCM)	*CACNB2*	rs41683753
	Infectious Diseases	Pathogenic Escherichia coli infection	*EZR*	rs110607520
		Hepatitis C	*CLDN18*	rs137294146
		Measles	*ADAR*	rs109349988
		Influenza A	*ADAR*	rs109349988
		Bacterial invasion of epithelial cells	*SHC1*	rs109349988
		HTLV-I infection	*PDGFA*	rs136287610
	Substance Dependence	Morphine addiction	*GNGT2, ADORA1*	rs136717249, rs41790889
		Nicotine addiction	*CHRNB2*	rs109349988
		Alcoholism	*GNGT2, SHC1*	rs136717249, rs109349988
	Cancers	Pathways in cancer	*CKS1B, PDGFA*	rs109349988, rs136287610
		Small cell lung cancer	*CKS1B*	rs109349988
		Glioma	*SHC1, PDGFA*	rs109349988, rs136287610
		Chronic myeloid leukemia	*SHC1*	rs109349988
		Transcriptional misregulation in cancers	*NGFR, PDGFA*	rs136717249, rs136287610
		Melanoma	*PDGFA*	rs136287610
		Prostate cancer	*PDGFA*	rs136287610

## Discussion

The use of the random forest approach as a first step, to filter candidate SNPs without taking into consideration a statistical model specification, is advantageous in genome-wide association studies, as long as little is known about candidate areas and the genetic architecture of the specific trait. Furthermore, the fact that results were obtained using two different strategies (Common SNPs and Highest 1% SNPs) and are very similar, provides reliability to the random forest methodology as can be seen in the previous study [43].

With the exception of four selected SNPs in the Highest 1% SNPs strategy (chr 12: rs136348926; chr 11: rs110833507; chr 2: rs42923911; chr 9: rs110025080), all other SNPs presented a fat-related QTL described in their chromosome region. Also, only one SNP on chr 3 (rs42021729) is not close to any described gene in the surrounding area (± 250kb) (Table 2).

In a previous genome-wide association study in Canchim, 100 SNPs on several chromosomes were considered the optimal set of SNPs to differentiate the 30 individuals with extreme phenotypes for backfat thickness. Among these SNPs, two haplotypes on chr 14 were genotyped and their association to the phenotype was validated in the whole population [33]. In the current study, even though SNPs from chr 14 were associated with backfat thickness by the random forest approach (in the Common SNP and Highest 1% SNP strategies, data not show), these SNPs were not selected in the stepwise regression model. Conflicting results and/or studies that cannot be replicated in the post-genomic area are not so uncommon [56-59], and these differences can be attributed to partially insufficient power, false-positive results, bias, sample size, and to differences in populations, controls, and methodologies [56-58], or true heterogeneity associations [56]. In these two GWA studies with Canchim, the base population is very similar, but the sample size and methodologies are not, which could explain the difference in the findings. A future option to help clarify the inconsistency in these findings would be to perform a meta-analysis, which combines data together to increase sample size and power, while reducing error risks [58,60].

Another outcome from this study and the previous one [33] is the possibility of including these SNPs in the development of a low density SNP (LD-SNP) panel for implementation of genomic selection in Canchim beef cattle. The most widespread strategy for developing small panels is by applying methods of variable selection to identify a diminutive set of SNPs that have good predictive power for the trait or breeding value [61]. The increase in accuracy of genomic breeding values obtained by using LD-SNP panels can be highly similar (around 90%) compared to the accuracies obtained by high density panels [62,63], at a more cost-effective price. Therefore, it is more likely to be adopted by farmers and the beef industry [64]. Furthermore, LD-SNP panels developed with SNPs selected on the basis of their effects perform better than LD-SNP panels with SNPs evenly spaced [62,63]. Importantly, SNPs identified in these studies need to undergo a prior validation in a population of animals which are not included in the population used for the SNP discovery (training population), enabling confidence in genomic predictions for future populations.

From the SNPs identified in this study, there were two on chr 10 (rs133046994, rs135638125) associated with backfat thickness, which together accounted for almost 12% of the dEBV variation (Table 2). These two SNPs are in the same chromosomal region as fat-related QTLs identified in previous studies [65,66], and they map to the same genes (*THSD4* - thrombospondin, type I, domain containing 4, and *LRRC49* - leucine-rich repeat-containing protein 49) thereby indicating *THSD4*, *LRRC49* and the surrounding areas as strong candidates for further investigations and validation. The *LRRC49* gene has been linked to breast cancer in humans, but very little is known about the biological function of the protein encoded by this gene [67].

The *THSD4* gene in *Bos taurus* and in *Homo sapiens* has a provisional status from RefSeq [68], which, by definition, supports that this gene is both transcribed and expressed. Further evidence for the annotation of this gene is given by its sequence identity in the UniGene database [52] when compared to orthologous sequences from *M. musculus* (95.1%), which has a validated status in RefSeq, and to *H. sapiens* (93.1%), suggesting a well-conserved homology of the *THSD4* gene in these species.

The *THSD4* gene encodes a protein with conserved disintegrin and metalloprotease domains, which it shares with the ADAM-TS1 protein family, and plays an import role in adipogenesis [69]. Previous studies have shown that this protein family interferes with the availability of differentiation-inducing or differentiation-inhibiting growth factors, either by modifying the extracellular matrix, affecting cell migration and adhesion, or by activating other pathways, which are key for regulating the differentiation of adipocytes, allowing their growth and expansion during adipogenesis [70].

The subcutaneous fat percentage QTL reported on chr 10 (Table 2) is from a Charolais × Holstein crossbred cattle population, and is described as highly significant with additive effects estimated to be 0.5 phenotypic standard deviation units [65]. The study also reveals that the Charolais allele was associated with higher fat levels.

The SNP on chr 1 (rs137294146) associated with backfat thickness is responsible for approximately 9.4% of the dEBV variation (Table 2). There is also a reported QTL for fat thickness over the 12th rib [29] and another for intramuscular fat percentage [71], indicating that there should be one or more genes in this area affecting fat metabolism. In the 500Kb window surrounding this SNP, three genes are annotated, *SOX14* (sex determining region Y – box 14), *CLDN18* (claudin 18), and *DZIP1L* (DAZ interacting protein 1-like). The *SOX14* gene seems to be involved in the regulation of embryonic development, whereas *CLDN18* belongs to a multigene family that encodes a tetraspanning membrane protein acting on components at tight junctions, but its regulatory mechanisms, and roles in physiology and pathology are still under investigation [72]. The *DZIP1L* gene encodes a zinc finger protein, but how it affects either adipogenesis or lipid metabolism has not been depicted from the current literature. Nonetheless, the functions of these gene products are still being elucidated.

The 500Kb window around the SNP on chr 3 (rs109349988) reveals many annotated genes, of which some have been reported as participating in lipid metabolism. For example, *PMVK* (phosphomevalonate kinase) catalyzes the conversion of mevalonate 5-phosphate with ATP to form mevalonate 5-diphosphate and ADP, which is one of the initial reactions involved in the cholesterol biosynthetic pathway [73]. Other proteins in this region include *ADAR* (adenosine deaminase, RNA-specific), which encodes an RNA-editing enzyme by site-specific deamination of adenosines, resulting in changes in protein function or gene expression. A study in humans was conducted that found ADAR enzymes were associated with serum triglyceride and adiponectin levels, abdominal circumference, and body mass index [74]. Interestingly, this region also contains *SHC1* (Src homology 2 domain containing – transforming protein 1) which has been reported as having a role in human obesity [75], and as being one of the mediators for regulating the insulin-like growth factor 1 (*IGF-1*) pathway, which plays a key role in regulating cell proliferation, differentiation and apoptosis [76]. Lastly, this region contains *ADAM15* (ADAM metallopeptidase domain 15), which belongs to the ADAM protein family previously discussed. These studies corroborate our findings and require further investigation to elucidate how these genes are affecting the deposition of subcutaneous fat in bovines.

The SNP associated with backfat thickness on chr 19 (rs136717249) is responsible for approximately 4.88% of the dEBV variance. This region contains the *PHOSPHO1* (phosphatase, orphan 1) gene, which encodes a phosphatase enzyme that has been implicated in the mineralization of the extracellular matrix, a key process for skeletal development [77]. The *PHOSPHO1* gene product has high activities toward phosphoethanolamine (PEA) and phosphocholine (PCho) [78], which are the main metabolites involved in the pathway for the formation of phosphatidylcholine and phosphatidylethanolamine [79]. These compounds are implicated in the metabolism of complex glycerolipids, prostaglandins, leukotrienes, glycosylphosphatidylinositol-anchors, and some amino acids, such as glycine, serine and threonine. Also included in this region is the *PHB* gene (prohibitin), which is thought to be involved in regulating cell proliferation, gene transcription, and apoptosis. In recent studies, deficient *PHB* activity in the liver has been associated with non-alcoholic steatohepatitis and obesity, although the mechanism remains unknown [80,81]. Other examples include the *IGF2BP1* (insulin-like growth factor 2 mRNA binding protein 1) gene, which encodes a protein that binds to the mRNAs of certain genes and regulates their translation. Lastly, the *GIP* (gastric inhibitory polypeptide, also known as the glucose-dependent insulinotropic polypeptide) gene has a known effect on stimulating the release of insulin from pancreatic β cells, but also has an insulin-like effect on adipocytes, suggesting that the *GIP* gene product enhances adipocyte glucose uptake, and that, at least in humans, it has an important role in the development of nutrition-induced obesity [82]. A recent study suggests that the *GIP* gene product has an effect on reducing free fatty acid release from adipose tissues, either by increasing reesterification or by inhibition of lipolysis [83]. Indeed, QTL studies reveal oleic acid content (OAC) and palmitoleic acid content (PAC) QTLs [84,85] in close proximity to the *GIP* gene in the bovine genome, which further suggests an association between this gene and free fatty acid processing.

The SNP rs134790147 on chr 13 also was associated with backfat thickness, and it is carrying 3.51% of the dEBV. Within this SNP region, a QTL for fat thickness over the 12th rib was found and described in an Angus population [29]. Also, a set of four genes are localized in the ±250kb window from the SNP position. The *CCDC7* gene (coiled-coil domain containing 7) seems to be associated with human cancer [86,87], and there is no information available for bovines. The *ARL5B* gene product (ADP-ribosylation factor-like 5B), also known as *ARL8*, belongs to a family of proteins that show similar structure to ADP-ribosylation factors (*ARFs* family). *ARL*s and *ARF*s belong to the RAS superfamily of small GTPases, which function as modulators of complex and diverse cellular processes [88,89], of which the most canonical are cell proliferation and differentiation. However, they are also involved in protein trafficking through the trans-Golgi network (TGN). The TGN has a central role in protein sorting and directs the transport of newly synthesized proteins to different transport vesicles [90-92], and also receives recycled molecules and extracellular materials by retrograde transport. Recently, it was observed that *ARL5B* enhances retrograde transport from endosomes to the TGN [93]. The *MGC152301* (uncharacterized LOC783682) and the *LOC524240* (Alk-like) genes do not have any available information in terms of function of their gene products, but both show the same two conserved domains: cd00112 (LDLa) and cd06263 (MAM) [94]. The LDLa is a low density lipoprotein receptor class A domain, that plays an important role in mammalian cholesterol metabolism, the protein receptor binds LDL and transports it into the cell by endocytosis [95]. The MAM is an extracellular domain that mediates protein-protein interactions, and is found in a variety of proteins, of which many are known to function in cell adhesion [96]. The remaining 16 SNPs, which were not described in detail here, accounted for 19.14% of dEBV variation for backfat thickness and, as seen in Table 2, most of them present some fat-related QTL described within their regions [29,65,66,85,97-99], and are of further interest for future investigations on how these SNPs can be influencing backfat thickness deposition in Canchim beef cattle.

## Conclusions

In this study, we were able to identify a set of SNPs that correlates with approximately 50% of the deregressed estimated breeding value variance for backfat thickness in Canchim beef cattle, which introduces the possibility of including these SNPs in the development of a low density SNP panel for future implementation of genomic selection program in Canchim beef cattle. We also have applied a new methodology using the Random Forest approach to identify novel gene candidates for improving backfat thickness in Canchim beef cattle. In addition, although this study used backfat thickness as a target trait, other analyses of this type have successfully used other traits, thereby supporting the random forest approach as a means of future investigations of livestock production traits. Lastly, some regions identified are not conspicuously associated with any specific genes. This suggests that they may be involved in as of yet unidentified regulatory functions of gene expression or processing. Given the intrinsic complexity of biochemical pathways, these regions and the genes within them merit a great deal of future investigations, specifically to how they correlate with backfat thickness deposition in Canchim beef cattle and to other breeds.

## Competing interests

The authors declare that they have no competing interests.

## Authors’ contributions

FBM: data analysis and interpretation, and primary author of the manuscript; RHH: SNP quality control analysis, SNP selection using Random Forest, and manuscript revision; MAM: data analysis, interpretation, and manuscript revision; AOL: data mining, interpretation; SLCM: data collection and analysis, DNA processing; MVGBS: experimental design, data analysis, interpretation, and manuscript revision; FFC: R script development, data analysis, and manuscript review; MMO: R script development; IU: R script development; SCMN: experimental design, preparation and handling of DNA samples for genotyping, and manuscript review; RRT: ultrasound measurements; MMA: experimental design; LCAR: experimental design, interpretation, and manuscript revision. All authors read and approved the final manuscript.

## References

[B1] Ferraz JBS Felício PE Production systems–an example from Brazil Meat Sci 2010 84 238 243 10.1016/j.meatsci.2009.06.006 20374781

[B2] Carvalho FA Lammoglia MA Simoes MJ Randel RD Breed affects thermoregulation and epithelial morphology in imported and native cattle subjected to heat stress J Anim Sci 1995 73 3570 3573 8655430 10.2527/1995.73123570x

[B3] Alencar MM Fraga AB Silva AM Adaptação de genótipos a ambientes tropicais: resistência à mosca-dos-chifres (Haematobia irritans, linnaeus) e ao carrapato (Boophilus microplus, CANESTRINI) Agrociencia 2005 IX 579 585

[B4] Silva AM Alencar MM Regitano LCA Oliveira MCS Infestação natural de fêmeas bovinas de corte por ectoparasitas na Região Sudeste do Brasil Revista Brasileira de Zootecnia 2010 39 1477 1482 10.1590/S1516-35982010000700012

[B5] Turner JW Genetic and biological aspects of Zebu adaptability J Anim Sci 1980 50 1201 1205 7400061 10.2527/jas1980.5061201x

[B6] Bianchini W Silveira AC Jorge AM Arrigoni MDB Martins CL Rodrigues É Hadlich JC Andrighetto C Efeito do grupo genético sobre as características de carcaça e maciez da carne fresca e maturada de bovinos superprecoces Revista Brasileira de Zootecnia 2007 36 2109 2117 10.1590/S1516-35982007000900022

[B7] Connor SFO Tatum JD Wulf DM Green RD Smith GC Genetic effects on beef tenderness in Bos indicus composite and Bos taurus cattle J Anim Sci 1997 75 1822 1830 9222838 10.2527/1997.7571822x

[B8] Whipple G Koohmaraie M Dikeman ME Crouse JD Hunt MC Klemm RD Evaluation of attributes that affect longissimus muscle tenderness in Bos taurus and Bos indicus cattle J Anim Sci 1990 68 2716 2728 2211401 10.2527/1990.6892716x

[B9] Highfill CM Esquivel-Font O Dikeman ME Kropf DH Tenderness profiles of ten muscles from F1 Bos indicus x Bos taurus and Bos taurus cattle cooked as steaks and roasts Meat Sci 2012 90 881 886 10.1016/j.meatsci.2011.11.022 22166845

[B10] Thrift FA Sanders JO Brown MA Brown AH Herring AD Riley DG DeRouen SM Holloway JW Wyatt WE Vann RC Chase CC Franke DE Cundiff LV Baker JF Review: Preweaning, Postweaning, and Carcass Trait Comparisons for Progeny Sired by Subtropically Adapted Beef Sire Breeds at Various US Locations Profl Anim S 2010 26 451 473

[B11] Chase CC Chenoweth PJ Larsen RE Olson TA Hammond AC Menchaca MA Randel RD Growth and reproductive development from weaning through 20 months of age among breeds of bulls in subtropical Florida Theriogenology 1997 47 723 745 10.1016/S0093-691X(97)00030-7 16728024

[B12] Nogueira GP Puberty in South American Bos indicus (Zebu) cattle Anim Reprod Sci 2004 82–83 361 372 15271466 10.1016/j.anireprosci.2004.04.007

[B13] Vianna AT Formação do gado Canchim pelo cruzamento charoles-zebu 1960 Rio de Janeiro: Ministério da Agricultura 48

[B14] Meirelles SL Alencar MM Oliveira HN Regitano LCA Efeitos de ambiente e estimativas de parâmetros genéticos para características de carcaça em bovinos da raça Canchim criados em pastagem Revista Brasileira de Zootecnia 2010 39 1437 1442 10.1590/S1516-35982010000700006

[B15] Rhoades R Ponce C Smith S Herring A TEDESCHI L Lunt DK Dean D Ribeiro F Choi C Riley D Sawyer J Evaluation of Growth-Based Predictions of Carcass Fat and Marbling at Slaughter Using Ultrasound Measurements Profl Anim S 2009 25 434 442

[B16] Utrera AR Van Vleck LD Heritability estimates for carcass traits of cattle: a review Genet Mol Res: GMR 2004 3 380 394 15614729

[B17] Lopes JS Rorato PRN Weber T Rodrigues RD Comin JG Dornelles M d A Metanálise para características de carcaça de bovinos de diferentes grupos genéticos Ciência Rural 2008 38 2278 2284 10.1590/S0103-8478200800080002923745029

[B18] Kemp DJ Herring WO Kaiser CJ Genetic and environmental parameters for steer ultrasound and carcass traits J Anim Sci 2002 80 1489 1496 12078728 10.2527/2002.8061489x

[B19] Crews DH Shannon NH Crews RE Kemp RA Weaning, yearling, and preharvest ultrasound measures of fat and muscle area in steers, bulls, and heifers J Anim Sci 2002 80 2817 2824 12462248 10.2527/2002.80112817x

[B20] Veneroni GB Meirelles SL Grossi DA Gasparin G Ibelli AMG Tizioto PC Oliveira HN Alencar MM Regitano LCA Prospecting candidate SNPs for backfat in Canchim beef cattle Genet Mol Res: GMR 2010 9 1997 2003 10.4238/vol9-4gmr788 20957603

[B21] Fortes MRS Curi RA Chardulo LAL Silveira AC Assumpção MEOD Visintin JA Oliveira HN Bovine gene polymorphisms related to fat deposition and meat tenderness Genet Mol Biol 2009 32 75 82 21637649 10.1590/S1415-47572009000100011 PMC3032970

[B22] Curi RA Krauskopf MM Hadlich JC Fortes MRS Vankan DM Silva JAIV de Oliveira HN da Mota MDS Candidate SNPs for carcass and meat traits in Nelore animals and in their crosses with Bos taurus Pesquisa Agropecuária Brasileira 2012 47 294 301 10.1590/S0100-204X201200020001923741531

[B23] De Carvalho TD TD Siqueira F de Torres Júnior RAA de Medeiros SR Feijó GLD de Souza Junior MD Blecha IMZ Soares CO Association of polymorphisms in the leptin and thyroglobulin genes with meat quality and carcass traits in beef cattle Revista Brasileira de Zootecnia 2012 41 2162 2168 10.1590/S1516-35982012001000004

[B24] Veneroni GB Meirelles SL De Oliveira HN De Alencar MM MM Gasparin G Association of CSSM066 and ILSTS011 microsatellite markers and thyroglobulin gene SNP with backfat in Canchim cattle Sci Agric 2012 69 1 5

[B25] Schenkel FS Miller SP Ye X Moore SS Nkrumah JD Li C Yu J Mandell IB Wilton JW Williams JL Association of single nucleotide polymorphisms in the leptin gene with carcass and meat quality traits of beef cattle J Anim Sci 2009 83 2009 2020 16100055 10.2527/2005.8392009x

[B26] Barendse W Haplotype analysis improved evidence for candidate genes for intramuscular fat percentage from a genome wide association study of cattle PLoS ONE 2011 6 e29601 10.1371/journal.pone.0029601 22216329 PMC3247274

[B27] Bolormaa S Porto Neto LR Zhang YD Bunch RJ Harrison BE Goddard ME Barendse W A genome-wide association study of meat and carcass traits in Australian cattle J Anim Sci 2011 89 2297 2309 10.2527/jas.2010-3138 21421834

[B28] Kim Y Ryu J Woo J Kim JB Kim CY Lee C Genome-wide association study reveals five nucleotide sequence variants for carcass traits in beef cattle Anim Genet 2011 42 361 365 10.1111/j.1365-2052.2010.02156.x 21749418

[B29] McClure MC Morsci NS Schnabel RD Kim JW Yao P Rolf MM McKay SD Gregg SJ Chapple RH Northcutt SL Taylor JF A genome scan for quantitative trait loci influencing carcass, post-natal growth and reproductive traits in commercial Angus cattle Anim Genet 2010 41 597 607 10.1111/j.1365-2052.2010.02063.x 20477797

[B30] Lindholm-Perry AK Sexten AK Kuehn LA Smith TPL King DA Shackelford SD Wheeler TL Ferrell CL Jenkins TG Snelling WM Freetly HC Association, effects and validation of polymorphisms within the NCAPG - LCORL locus located on BTA6 with feed intake, gain, meat and carcass traits in beef cattle BMC Genet 2011 12 103 22168586 10.1186/1471-2156-12-103 PMC3287254

[B31] Soma Y Uemoto Y Sato S Shibata T Kadowaki H Kobayashi E Suzuki K Genome-wide mapping and identification of new quantitative trait loci affecting meat production, meat quality, and carcass traits within a Duroc purebred population J Anim Sci 2011 89 601 608 10.2527/jas.2010-3119 21097684

[B32] Peters SO Kizilkaya K Garrick DJ Fernando RL Reecy JM Weaber RL Silver GA Thomas MG Bayesian genome wide association analyses of growth and yearling ultrasound measures of carcass traits in Brangus heifers J Anim Sci 2012 90 3398 3409 23038745 10.2527/jas.2012-4507

[B33] Veneroni-Gouveia G Meirelles SL Grossi DA Santiago AC Sonstegard TS Yamagishi MEB Matukumalli LK Coutinho LL Alencar MM Oliveira HN Regitano LCA Whole-genome analysis for backfat thickness in a tropically adapted, composite cattle breed from Brazil Anim Genet 2012 43 518 524 10.1111/j.1365-2052.2011.02286.x 22497247

[B34] Gill JL Bishop SC McCorquodale C Williams JL Wiener P Association of selected SNP with carcass and taste panel assessed meat quality traits in a commercial population of Aberdeen Angus-sired beef cattle Genet, Sel, Evol: GSE 2009 41 36 10.1186/1297-9686-41-36 PMC2714298 19555501

[B35] Porto Neto LR Bunch RJ Harrison BE Barendse W Variation in the XKR4 gene was significantly associated with subcutaneous rump fat thickness in indicine and composite cattle Anim Genet 2012 43 785 789 10.1111/j.1365-2052.2012.02330.x 22497494

[B36] Hoh J Ott J Mathematical multi-locus approaches to localizing complex human trait genes Nat Rev Genet 2003 4 701 709 12951571 10.1038/nrg1155

[B37] Balding DJ A tutorial on statistical methods for population association studies Nat Rev Genet 2006 7 781 791 10.1038/nrg1916 16983374

[B38] Long N Gianola D Rosa GJM Weigel KA Avendaño S Machine learning classification procedure for selecting SNPs in genomic selection: application to early mortality in broilers J Anim Breed Genet 2007 124 377 389 10.1111/j.1439-0388.2007.00694.x 18076475

[B39] Breiman L Random Forests Mach Learn 2001 45 5 32 10.1023/A:1010933404324

[B40] Breiman L Classification and regression trees 1984 Belmont, Calif: Wadsworth International Group 358

[B41] Breiman L Bagging Predictors Mach Learn 1996 140 123 140

[B42] Ho TK The random subspace method for constructing decision forests IEEE Trans Pattern Anal Mach Intell 1998 20 832 844 10.1109/34.709601

[B43] Goldstein BA Hubbard AE Cutler A Barcellos LF An application of Random Forests to a genome-wide association dataset: methodological considerations & new findings BMC Genet 2010 11 49 20546594 10.1186/1471-2156-11-49 PMC2896336

[B44] Boldman KG Kriese LA Vleck V Tassell V Kachman SD A Manual for Use of MTDFREML. A Set of Programs to Obtain Estimates of Variances and Covariances 1995 [Draft]. Washington. DC: U.S. Department of Agriculture, Agricultural Research Service

[B45] Garrick DJ Taylor JF Fernando RL Deregressing estimated breeding values and weighting information for genomic regression analyses Genet, Sel, Evol: GSE 2009 41 55 10.1186/1297-9686-41-55 PMC2817680 20043827

[B46] Liaw A Wiener M Classification and Regression by randomForest R News 2002 2 18 22

[B47] R Development Core Team R: A Language and Environment for Statistical Computing R Found Stat Comput 2011 1 409

[B48] SAS Institute Inc SAS/STAT Software 2011 SAS Institute Inc: Version 9.3. Cary NC

[B49] Scheet P Stephens M A fast and flexible statistical model for large-scale population genotype data: applications to inferring missing genotypes and haplotypic phase Am J Human Genet 2006 78 629 644 10.1086/502802 16532393 PMC1424677

[B50] Barrett JC Fry B Maller J Daly MJ Haploview: analysis and visualization of LD and haplotype maps Bioinformatics (Oxford, England) 2005 21 263 265 10.1093/bioinformatics/bth457 15297300

[B51] Cattle Genome UMD3.1 http://www.animalgenome.org/cgi-bin/gbrowse/bovine/

[B52] Geer LY Marchler-Bauer A Geer RC Han L He J He S Liu C Shi W Bryant SH The NCBI BioSystems database Nucleic Acids Res 2010 38 D492 D496 10.1093/nar/gkp858 19854944 PMC2808896

[B53] Kanehisa M Goto S KEGG: kyoto encyclopedia of genes and genomes Nucleic Acids Res 2000 28 27 30 10.1093/nar/28.1.27 10592173 PMC102409

[B54] Kanehisa M Goto S Sato Y Furumichi M Tanabe M KEGG for integration and interpretation of large-scale molecular data sets Nucleic Acids Res 2012 40 D109 D114 10.1093/nar/gkr988 22080510 PMC3245020

[B55] Anderson MJ Permutation tests for univariate or multivariate analysis of variance and regression Can J Fish Aquatic Sci 2001 58 626 639 10.1139/f01-004

[B56] Colhoun H Mckeigue P Smith G Problems of reporting genetic associations with complex outcomes Lancet 2003 361 865 872 10.1016/S0140-6736(03)12715-8 12642066

[B57] Sun Q Song K Shen X Cai Y The association between KCNQ1 gene polymorphism and type 2 diabetes risk: a meta-analysis PLoS ONE 2012 7 e48578 10.1371/journal.pone.0048578 23133642 PMC3487731

[B58] Song Q Zhu B Hu W Cheng L Gong H Xu B Zheng X Zou L Zhong R Duan S Chen W Rui R Wu J Miao X A common SMAD7 variant is associated with risk of colorectal cancer: evidence from a case–control study and a meta-analysis PLoS ONE 2012 7 e33318 10.1371/journal.pone.0033318 22457752 PMC3310071

[B59] Luo Y Jin C Ling Z Mou X Zhang Q Xiang C Association study of IL28B: rs12979860 and rs8099917 polymorphisms with SVR in patients infected with chronic HCV genotype 1 to PEG-INF/RBV therapy using systematic meta-analysis Gene 2013 513 292 296 10.1016/j.gene.2012.10.030 23142377

[B60] Pabalan NA Meta-analysis in cancer genetics Asian Pac J Cancer Prev: APJCP 2010 11 33 38 20593927

[B61] Habier D Fernando RL Dekkers JCM Genomic selection using low-density marker panels Genetics 2009 182 343 353 10.1534/genetics.108.100289 19299339 PMC2674831

[B62] Moser G Khatkar MS Hayes BJ Raadsma HW Accuracy of direct genomic values in Holstein bulls and cows using subsets of SNP markers Gen, Sel, Evol: GSE 2010 42 37 10.1186/1297-9686-42-37 PMC2964565 20950478

[B63] Zhang Z Ding X Liu J Zhang Q De Koning D-J Accuracy of genomic prediction using low-density marker panels J Dairy Sci 2011 94 3642 3650 10.3168/jds.2010-3917 21700054

[B64] Garrick DJ The nature, scope and impact of genomic prediction in beef cattle in the United States Gen, Sel, Evol: GSE 2011 43 17 10.1186/1297-9686-43-17 PMC3107171 21569623

[B65] Gutiérrez-Gil B Williams JL Homer D Burton D Haley CS Wiener P Search for quantitative trait loci affecting growth and carcass traits in a cross population of beef and dairy cattle J Anim Sci 2009 87 24 36 18791160 10.2527/jas.2008-0922

[B66] Casas E Shackelford SD Keele JW Koohmaraie M Smith TPL Stone RT Detection of quantitative trait loci for growth and carcass composition in cattle J Anim Sci 2003 81 2976 2983 14677852 10.2527/2003.81122976x

[B67] De Souza Santos E De Bessa SA Netto MM Nagai MA Silencing of LRRC49 and THAP10 genes by bidirectional promoter hypermethylation is a frequent event in breast cancer Int J Oncol 2008 33 25 31 18575747

[B68] Pruitt KD Tatusova T Brown GR Maglott DR NCBI Reference Sequences (RefSeq ): current status, new features and genome annotation policy Nucleic Acids Res 2012 40 130 135 10.1093/nar/gkr1079 PMC3245008 22121212

[B69] McDaniel AH Li X Tordoff MG Bachmanov AA Reed DR A locus on mouse Chromosome 9 (Adip5) affects the relative weight of the gonadal but not retroperitoneal adipose depot Mamm Genome 2006 17 1078 1092 10.1007/s00335-006-0055-1 17103052 PMC1698868

[B70] Lilla J Stickens D Werb Z Metalloproteases and adipogenesis: a weighty subject Am J Pathol 2002 160 1551 1554 10.1016/S0002-9440(10)61100-5 12000705 PMC1850859

[B71] Gutiérrez-Gil B Wiener P Nute GR Burton D Gill JL Wood JD Williams JL Detection of quantitative trait loci for meat quality traits in cattle Anim Genet 2008 39 51 61 10.1111/j.1365-2052.2007.01682.x 18254735

[B72] Lal-Nag M Morin PJ The claudins Genome Biol 2009 10 235 10.1186/gb-2009-10-8-235 19706201 PMC2745760

[B73] Herdendorf TJ Miziorko HM Functional evaluation of conserved basic residues in human phosphomevalonate kinase Biochemistry 2007 46 11780 11788 10.1021/bi701408t 17902708 PMC2530820

[B74] Oguro R Kamide K Katsuya T Akasaka H Sugimoto K Congrains A Arai Y Hirose N Saitoh S Ohishi M Miura T Rakugi H A single nucleotide polymorphism of the adenosine deaminase, RNA-specific gene is associated with the serum triglyceride level, abdominal circumference, and serum adiponectin concentration Exp Gerontol 2012 47 183 187 10.1016/j.exger.2011.12.004 22210125

[B75] Feigelson HS Teras LR Diver WR Tang W Patel AV Stevens VL Calle EE Thun MJ Bouzyk M Genetic variation in candidate obesity genes ADRB2, ADRB3, GHRL, HSD11B1, IRS1, IRS2, and SHC1 and risk for breast cancer in the Cancer Prevention Study II Breast Cancer Res: BCR 2008 10 R57 10.1186/bcr2114 18611262 PMC2575528

[B76] Wagner K Hemminki K Grzybowska E Klaes R Butkiewicz D Pamula J Pekala W Zientek H Mielzynska D Siwinska E Försti A The insulin-like growth factor-1 pathway mediator genes: SHC1 Met300Val shows a protective effect in breast cancer Carcinogenesis 2004 25 2473 2478 10.1093/carcin/bgh263 15308584

[B77] Huesa C Yadav MC Finnilä MAJ Goodyear SR Robins SP Tanner KE Aspden RM Millán JL Farquharson C PHOSPHO1 is essential for mechanically competent mineralization and the avoidance of spontaneous fractures Bone 2011 48 1066 1074 10.1016/j.bone.2011.01.010 21272676 PMC3078982

[B78] Roberts SJ Stewart AJ Sadler PJ Farquharson C Human PHOSPHO1 exhibits high specific phosphoethanolamine and phosphocholine phosphatase activities Biochem J 2004 382 59 65 10.1042/BJ20040511 15175005 PMC1133915

[B79] Walkey CJ Donohue LR Bronson R Agellon LB Vance DE Disruption of the murine gene encoding phosphatidylethanolamine N-methyltransferase Proc Nat Acad Sci U S Am 1997 94 12880 12885 10.1073/pnas.94.24.12880 PMC24232 9371769

[B80] Theiss AL Sitaraman SV The role and therapeutic potential of prohibitin in disease Biochimica et Biophysica Acta 1813 2011 1137 1143 10.1016/j.bbamcr.2011.01.033 PMC3370678 21296110

[B81] Sánchez-Quiles V Santamaría E Segura V Sesma L Prieto J Corrales FJ Prohibitin deficiency blocks proliferation and induces apoptosis in human hepatoma cells: molecular mechanisms and functional implications Proteomics 2010 10 1609 1620 10.1002/pmic.200900757 20186755

[B82] Song DH Getty-Kaushik L Tseng E Simon J Corkey BE Wolfe MM Glucose-dependent insulinotropic polypeptide enhances adipocyte development and glucose uptake in part through Akt activation Gastroenterology 2007 133 1796 1805 10.1053/j.gastro.2007.09.005 18054552 PMC2185546

[B83] Gögebakan Ö Andres J Biedasek K Mai K Kühnen P Krude H Isken F Rudovich N Osterhoff MA Kintscher U Nauck M Pfeiffer AFH Spranger J Glucose-dependent insulinotropic polypeptide reduces fat-specific expression and activity of 11β-hydroxysteroid dehydrogenase type 1 and inhibits release of free fatty acids Diabetes 2012 61 292 300 10.2337/db10-0902 22179810 PMC3266397

[B84] Morris CA Cullen NG Glass BC Hyndman DL Manley TR Hickey SM McEwan JC Pitchford WS Bottema CDK Lee MAH Fatty acid synthase effects on bovine adipose fat and milk fat Mamm Genome 2007 18 64 74 10.1007/s00335-006-0102-y 17242864

[B85] Morris CA Bottema CDK Cullen NG Hickey SM Esmailizadeh AK Siebert BD Pitchford WS Quantitative trait loci for organ weights and adipose fat composition in Jersey and Limousin back-cross cattle finished on pasture or feedlot Anim Genetics 2010 41 589 596 10.1111/j.1365-2052.2010.02058.x 20477785

[B86] Shen Y-M Arbman G Sandström P Gullstrand P Wei Y-Q Zhang H Rosell J Olsson B Peng F Yang H-S Wang C-T Sun X-F Novel gene hBiot2 is an independent prognostic factor in colorectal cancer patients Oncol Rep 2012 27 376 382 22024937 10.3892/or.2011.1521

[B87] Shen YM He X Deng HX Xie YP Wang CT Wei YQ Zhao X Overexpression of the hBiot2 gene is associated with development of human cervical cancer Oncol Rep 2011 25 75 80 21109960

[B88] Macara IG Lounsbury KM Richards SA McKiernan C Bar-Sagi D The Ras superfamily of GTPases FASEB J 1996 10 625 630 8621061 10.1096/fasebj.10.5.8621061

[B89] Wennerberg K Rossman KL Der CJ The Ras superfamily at a glance J Cell Sci 2005 118 843 846 10.1242/jcs.01660 15731001

[B90] Griffiths G Simons K The trans Golgi network: sorting at the exit site of the Golgi complex Science (New York, N.Y.) 1986 234 438 443 10.1126/science.2945253 2945253

[B91] Gu F Crump CM Thomas G Trans-Golgi network sorting Cell Mol Life Sci 2001 58 1067 1084 10.1007/PL00000922 11529500 PMC1424219

[B92] Bonifacino JS Rojas R Retrograde transport from endosomes to the trans-Golgi network. Nature reviews Mol Cell Biol 2006 7 568 579 10.1038/nrm1985 16936697

[B93] Houghton FJ Bellingham SA Hill AF Bourges D Ang DKY Gemetzis T Gasnereau I Gleeson PA Arl5b is a Golgi-localised small G protein involved in the regulation of retrograde transport Exp Cell Res 2012 318 464 477 10.1016/j.yexcr.2011.12.023 22245584

[B94] Marchler-Bauer A Zheng C Chitsaz F Derbyshire MK Geer LY Geer RC Gonzales NR Gwadz M Hurwitz DI Lanczycki CJ Lu F Lu S Marchler GH Song JS Thanki N Yamashita RA Zhang D Bryant SH CDD: conserved domains and protein three-dimensional structure Nucleic Acids Res 2013 41 D348 D352 10.1093/nar/gks1243 23197659 PMC3531192

[B95] Huang W Dolmer K Gettins PG NMR solution structure of complement-like repeat CR8 from the low density lipoprotein receptor-related protein J Biol Chem 1999 274 14130 14136 10.1074/jbc.274.20.14130 10318830

[B96] Aricescu AR Hon W-C Siebold C Lu W Van der Merwe PA Jones EY Molecular analysis of receptor protein tyrosine phosphatase mu-mediated cell adhesion EMBO J 2006 25 701 712 10.1038/sj.emboj.7600974 16456543 PMC1383555

[B97] Yokouchi K Mizoguchi Y Watanabe T Iwamoto E Sugimoto Y Takasuga A Identification of a 3.7-Mb region for a marbling QTL on bovine chromosome 4 by identical-by-descent and association analysis Anim Genetics 2009 40 945 951 10.1111/j.1365-2052.2009.01956.x 19781039

[B98] Casas E Stone RT Keele JW Shackelford SD Kappes SM Koohmaraie M A comprehensive search for quantitative trait loci affecting growth and carcass composition of cattle segregating alternative forms of the myostatin gene J Anim Sci 2001 79 854 860 11325189 10.2527/2001.794854x

[B99] Imumorin IG Kim E-H Lee Y-M De Koning D-J Van Arendonk JA De Donato M Taylor JF Kim J-J Genome scan for parent-of-origin QTL effects on bovine growth and carcass traits Front Genetics 2011 2 44 10.3389/fgene.2011.00044 PMC3268597 22303340

